# Why Do Cells Contain Thousands of Lipid Species? Toward an Integrated Framework for Lipid Diversity in Biological Membranes

**DOI:** 10.3390/ijms27094089

**Published:** 2026-05-02

**Authors:** Kyung-Hee Kim, Byong Chul Yoo

**Affiliations:** 1 Department of Applied Chemistry, School of Science and Technology, Kookmin University, Seoul 02707, Republic of Korea; kyungheekim@kookmin.ac.kr; 2 Antibody Research Institute, Kookmin University, Seoul 02707, Republic of Korea; 3 Diagnostic Research Team, InnoBation Bio R&D Center, Seoul 03929, Republic of Korea

**Keywords:** lipid diversity, lipidome, biological membranes, membrane organization, lipid–protein interactions, lipid signaling, lipid metabolism, membrane evolution

## Abstract

Cells contain an unexpectedly large diversity of lipid molecules. Modern lipidomics studies have revealed that even a single cell type can harbor hundreds to thousands of distinct lipid species that differ in headgroup structure, acyl chain length, and degree of unsaturation. While this remarkable diversity is now well established, its biological significance remains incompletely understood. Why do cells maintain such complex lipidomes? In this review, we examine several conceptual frameworks that may help explain the origin and functional significance of lipid diversity. First, the physical properties of biological membranes impose constraints on lipid composition, as variations in lipid structure influence membrane fluidity, curvature, thickness, and phase behavior. Second, lipids can regulate membrane protein function through specific interactions and through the physical environment of the lipid bilayer. Third, lipid metabolism generates signaling molecules that participate in diverse regulatory pathways. Fourth, lipid metabolic networks continuously remodel membrane composition, producing dynamic lipidomes that can adapt to physiological conditions. Finally, evolutionary processes have shaped membrane lipid composition across different domains of life, suggesting that lipid diversity may reflect long-term adaptation to functional and environmental constraints. Taken together, these perspectives suggest that lipid diversity is unlikely to be a simple byproduct of metabolism. Instead, the cellular lipidome may emerge from the interplay of membrane biophysics, metabolic network architecture, protein regulation, and evolutionary pressures. Understanding why cells contain thousands of lipid species therefore represents an important challenge for modern cell biology and may reveal fundamental principles governing the organization of biological membranes.

## 1. Introduction: The Lipid Diversity Problem

Lipids are among the most abundant molecular components of living cells and form the structural foundation of biological membranes. In addition to providing a permeability barrier that separates cellular compartments, membrane lipids contribute to a wide range of cellular processes, including membrane trafficking, signal transduction, and energy metabolism [1,2,3]. Over the past two decades, advances in mass spectrometry-based lipidomics have revealed that cellular membranes contain an unexpectedly large diversity of lipid molecules [4,5,6,7,8,9,10,11]. Even a single cell type can harbor hundreds to thousands of distinct lipid species that differ in headgroup structure, acyl chain length, and degree of unsaturation [3,12,13,14,15,16]. While this remarkable molecular diversity is now well documented, its biological significance remains unclear.

### Why Do Cells Maintain Such Complex Lipidomes Containing Thousands of Distinct Lipid Species?

One possible explanation is that lipid diversity simply reflects the biochemical flexibility of lipid metabolic pathways. Lipid biosynthesis involves numerous enzymatic reactions that combine different fatty acids with various backbone molecules and headgroups, naturally generating a large number of possible molecular species [17,18].

From this perspective, lipid diversity could be interpreted as a metabolic byproduct rather than a biologically meaningful feature. Lipid compositions are tightly regulated and vary across cell types, tissues, and organelles. Specific lipid molecules are known to regulate membrane protein function and signaling pathways, and lipidomes change dynamically in response to environmental conditions and physiological states. These observations suggest that lipid diversity may play important roles in membrane organization and cellular regulation.

However, several observations suggest that lipid diversity may have functional significance. First, the lipid composition of membranes is highly regulated and varies between cell types, tissues, and organelles [1,13,19]. Second, specific lipid molecules are known to regulate membrane proteins and signaling pathways [20,21,22]. Third, lipid compositions change dynamically in response to environmental conditions and physiological states [6,23]. These observations suggest that lipid diversity may not be merely accidental but could reflect fundamental principles of membrane organization and cellular regulation.

Understanding why cells maintain such a diverse lipid repertoire therefore represents an important challenge in modern cell biology. Several conceptual frameworks have been proposed to explain lipid diversity in biological membranes [24]. Addressing this question requires integrating perspectives from membrane biophysics, metabolism, cell signaling, and evolution.

**Figure 1:**
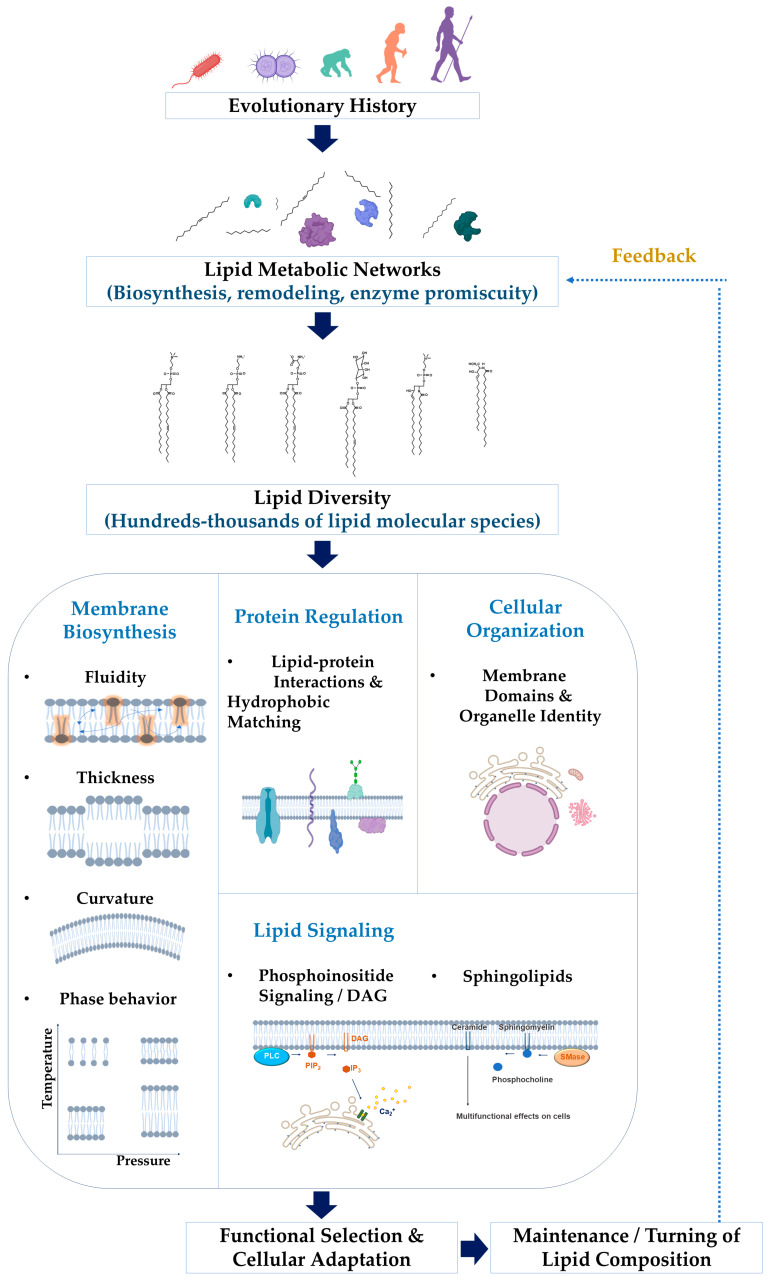
Integrated framework linking lipid metabolic networks, lipid diversity, and membrane functions. Lipid metabolic processes and evolutionary constraints generate diverse lipid species that differ in headgroup structure, acyl chain length, and degree of unsaturation. This diversity contributes to membrane physical properties, regulates membrane protein activity, and supports lipid-mediated signaling pathways. Feedback mechanisms further shape lipid composition through metabolic remodeling and cellular adaptation.

In this review, we examine several conceptual frameworks that may help explain why cells maintain large numbers of lipid species. These include the physical constraints of membrane structure, lipid–protein interactions, lipid-mediated signaling, metabolic network dynamics, and evolutionary adaptation. Together, these perspectives suggest that lipid diversity may emerge from the interplay of membrane biophysics, metabolic network architecture, protein regulation, and evolutionary pressures (Figure 1). The mechanistic details of lipid-mediated signaling and metabolic feedback are discussed in later sections.

## 2. The Scale of Lipid Diversity

The diversity of cellular lipids becomes apparent when lipidomes are analyzed using high-resolution mass spectrometry. Comprehensive lipidomic studies have revealed that even relatively simple eukaryotic cells contain hundreds of distinct lipid species, while mammalian cells may contain thousands [3,5,7,11,12,25]. For example, large-scale analyses of the human plasma lipidome have identified hundreds of lipid species across multiple lipid classes, including glycerophospholipids, sphingolipids, sterols, and neutral lipids [6]. Similar levels of diversity have been reported in yeast, plants, and bacteria, although the specific lipid compositions vary across organisms [12,26,27,28].

This diversity arises from several structural dimensions of lipid molecules. First, lipids can differ in their headgroup composition, which determines the chemical and electrostatic properties of the membrane surface. Common headgroups include phosphatidylcholine, phosphatidylethanolamine, phosphatidylserine, and phosphatidylinositol [1,13]. Second, lipids vary in the length and saturation of their acyl chains. Fatty acids can differ in chain length and number of double bonds, generating a wide range of molecular species within each lipid class [3,23]. Third, lipids can undergo remodeling reactions that alter their acyl chains after synthesis. These remodeling pathways, often referred to as the Lands cycle, further expand lipid diversity within cellular membranes [17,18].

Together, these structural variables create an enormous combinatorial space of possible lipid molecules. Yet cells do not randomly populate this space. Instead, lipid compositions are tightly regulated and display characteristic patterns that differ between organelles and physiological conditions [1,3,19]. These observations suggest that lipid diversity may reflect functional constraints rather than purely biochemical chance. Recent lipidomic studies indicate that mammalian cells can contain more than 1000 distinct lipid species, with abundance distributions spanning several orders of magnitude [6,25]. Importantly, these lipidomes are not randomly distributed but exhibit structured abundance patterns, with a small number of dominant species and a long tail of low-abundance lipids. This quantitative organization suggests that lipid diversity reflects underlying metabolic network architecture and regulatory constraints rather than purely stochastic variation. Several conceptual frameworks have been proposed to explain why cells maintain such complex lipidomes [24] (Table 1).

## 3. Hypothesis 1: Physical Requirements of Membrane Organization

One of the most widely discussed explanations for lipid diversity is that it arises from the physical requirements of membrane structure and dynamics. Biological membranes are complex two-dimensional fluids whose properties depend strongly on lipid composition. Variations in acyl chain saturation, chain length, and headgroup interactions influence membrane fluidity, thickness, curvature, and phase behavior [1,29,30,40,41].

For example, unsaturated lipids tend to increase membrane fluidity, whereas saturated lipids and cholesterol promote more ordered membrane states [30,31]. These differences can influence the formation of membrane domains, including liquid-ordered and liquid-disordered phases, which may organize membrane proteins and signaling complexes [31,42,43,44]. Importantly, the classical concept of lipid rafts as stable, micrometer-scale domains has been substantially revised. Recent experimental and computational studies indicate that membrane heterogeneity arises from highly dynamic, nanoscale assemblies driven by transient lipid–lipid and lipid–protein interactions. These nanodomains are typically small (10–200 nm), short-lived, and strongly dependent on local lipid composition, cholesterol content, and protein organization [41,42]. In this modern view, cholesterol and sphingolipids promote local ordering and domain formation, but these structures are not static entities; rather, they represent emergent and continuously fluctuating features of biological membranes [31]. This reinterpretation has important implications for lipid diversity. A heterogeneous lipid composition may be required to support the formation of multiple, coexisting membrane environments, enabling spatial and temporal regulation of membrane proteins and signaling processes at the nanoscale.

Lipid diversity may therefore allow cells to finely tune the physical properties of membranes. By adjusting lipid composition, cells can regulate membrane thickness, elasticity, and curvature, enabling processes such as vesicle trafficking, membrane fusion, and organelle formation [45,46,47,48]. From this perspective, lipid diversity reflects the physical demands of membrane architecture rather than the specific biochemical functions of individual lipid species. Lipid diversity arises from multiple structural variables, including differences in headgroups, acyl chain length, and degree of unsaturation [3,29,30] (Table 2).

## 4. Lipid–Protein Interactions as a Source of Functional Diversity

Biological membranes are not composed solely of lipids. They also contain a large number of membrane proteins that perform essential cellular functions, including signal transduction, transport, and energy conversion. The activity of many of these proteins depends strongly on the lipid environment in which they are embedded [20,33,45,49].

Traditionally, membrane lipids were viewed primarily as a passive solvent that provides a structural matrix for membrane proteins. In this view, the specific identities of lipid molecules were considered relatively unimportant as long as the membrane maintained appropriate physical properties such as fluidity and thickness. However, increasing evidence suggests that lipids can interact with membrane proteins in more specific ways [50].

### 4.1. Annular and Non-Annular Lipids

Membrane proteins are typically surrounded by a shell of lipid molecules known as annular lipids. These lipids form the immediate lipid environment around the protein and can influence its conformational dynamics and stability [20,32,51,52]. In addition to annular lipids, many membrane proteins contain binding pockets that accommodate specific lipid molecules. These non-annular lipids can bind tightly to membrane proteins and sometimes play structural or regulatory roles [21,53]. High-resolution structural studies have increasingly revealed lipid molecules bound to membrane proteins in well-defined positions [54,55]. In many cases, these lipids appear to stabilize particular conformational states of the protein or modulate its activity [21,53].

### 4.2. Hydrophobic Matching and Membrane Thickness

Another mechanism linking lipid composition to protein function involves hydrophobic matching between membrane proteins and the surrounding lipid bilayer. The transmembrane regions of membrane proteins contain hydrophobic segments whose length typically matches the hydrophobic thickness of the lipid bilayer. If the bilayer thickness differs substantially from the hydrophobic length of the protein, energetic penalties arise that can alter protein conformation or induce local membrane deformation [32,33,56]. Variations in lipid composition can therefore modulate protein function by altering membrane thickness and elastic properties [57]. In this sense, lipid diversity provides a means of adjusting the physical environment in which membrane proteins operate.

### 4.3. Specific Lipid Regulation of Membrane Proteins

Beyond these general physical effects, certain lipid species appear to regulate membrane proteins in highly specific ways. For example, cholesterol has been shown to interact directly with several classes of membrane proteins, including G protein-coupled receptors (GPCRs) and ion channels [45,58,59,60]. These interactions can influence receptor stability, ligand binding, and signaling activity. Similarly, phosphoinositides such as phosphatidylinositol 4,5-bisphosphate (PIP_2_) are known to regulate a variety of membrane proteins, including ion channels and cytoskeletal regulators [34,35]. In these cases, the lipid molecule functions almost like a regulatory ligand that modulates protein activity. Another striking example involves cardiolipin, a mitochondrial phospholipid that plays a crucial role in stabilizing protein complexes of the respiratory chain [61,62]. The presence of cardiolipin is essential for the proper assembly and function of several mitochondrial membrane protein complexes.

### 4.4. Implications for Lipid Diversity

These observations raise an intriguing possibility: lipid diversity may be required because different membrane proteins depend on distinct lipid environments. If membrane proteins interact selectively with particular lipid species, then maintaining a diverse lipid repertoire could allow cells to regulate many different proteins simultaneously [63]. In this view, lipid diversity would provide a flexible molecular toolkit for modulating membrane protein function across different cellular contexts. Such a model would also explain why lipid compositions differ between organelles and cell types. Different membrane proteins may impose distinct lipid requirements, leading to the evolution of specialized lipid environments in different cellular compartments.

## 5. Lipid Signaling and the Possibility of Information Encoding

In addition to their structural roles in membranes, many lipids participate directly in cellular signaling. Unlike proteins or nucleic acids, lipids were historically not considered major information-carrying molecules. However, increasing evidence suggests that lipid molecules can function as highly specific regulators of signaling pathways [64,65,66]. A wide variety of signaling lipids have been identified, including phosphoinositides, diacylglycerol (DAG), sphingolipids, and eicosanoids. Importantly, these signaling systems do not rely on a single lipid species but instead involve multiple structurally related molecules that differ in headgroup modification, acyl chain composition, and subcellular localization. This combinatorial diversity enables fine-tuned and context-dependent regulation of signaling pathways, suggesting that lipid diversity is not merely a byproduct of metabolism but a functional requirement for encoding regulatory specificity in cellular signaling networks [22,36,37,67,68,69,70,71,72]. In this sense, lipid signaling systems illustrate how molecular diversity can expand the regulatory capacity of membranes beyond what would be achievable with a limited set of lipid species. Importantly, these signaling processes are often tightly linked to membrane composition and lipid metabolism. Numerous lipid molecules have been shown to regulate cellular signaling pathways through diverse mechanisms [35,54,55,56,57,58,59,60,61,62,63,64,65,66,73,74] (Table 3). Representative examples of lipid-mediated signaling include phosphoinositide pathways, in which phosphatidylinositol 4,5-bisphosphate (PIP_2_) is hydrolyzed by phospholipase C to generate diacylglycerol (DAG) and inositol trisphosphate (IP_3_), leading to downstream signaling events such as intracellular Ca^2+^ release [35,75]. Similarly, sphingolipid metabolism can produce signaling molecules such as ceramide through the action of sphingomyelinase, linking membrane lipid composition to stress signaling pathways [22,76].

### 5.1. Phosphoinositides as Spatial Signaling Lipids

One of the most extensively studied lipid signaling systems involves phosphoinositides, a family of phosphatidylinositol derivatives that differ in the phosphorylation pattern of the inositol headgroup. Although phosphoinositides represent only a small fraction of total membrane lipids, they play critical roles in signaling and membrane dynamics. Different phosphoinositide species are enriched in distinct cellular membranes and organelles, where they recruit specific effector proteins through lipid-binding domains such as PH, FYVE, and PX domains [34,35,65,66]. For example, phosphatidylinositol 4,5-bisphosphate (PIP_2_) is concentrated at the plasma membrane and regulates processes such as cytoskeletal organization, endocytosis, and ion channel activity [34,35,65]. Other phosphoinositides define membrane identity in intracellular compartments such as the Golgi apparatus and endosomes [34,66]. These observations illustrate how a relatively small set of lipid molecules can generate spatially organized signaling systems within cellular membranes.

### 5.2. Lipid-Derived Second Messengers

Many lipid molecules also function as second messengers generated through enzymatic modification of membrane phospholipids. One classic example is diacylglycerol (DAG), which is produced by phospholipase C-mediated cleavage of phosphatidylinositol lipids. DAG activates members of the protein kinase C (PKC) family and plays a central role in receptor-mediated signaling pathways [22,68,70]. Similarly, sphingolipid metabolism generates signaling molecules such as ceramide and sphingosine-1-phosphate (S1P). These lipids regulate diverse cellular processes, including apoptosis, proliferation, inflammation, and cell migration [22,36,71,77,78]. In these cases, lipid molecules act not only as membrane components but also as transient signaling intermediates linking extracellular signals to intracellular responses.

### 5.3. Lipid Mediators in Intercellular Communication

Some lipids function as diffusible signaling molecules that mediate communication between cells and tissues. A well-known example is the family of eicosanoids, which are derived from arachidonic acid released from membrane phospholipids. These molecules play key roles in inflammation, immune responses, and vascular physiology [37,67,72]. Other lipid mediators, such as lysophosphatidic acid (LPA) and sphingosine-1-phosphate, can activate specific G protein-coupled receptors and regulate processes including cell migration and tissue remodeling [36,79,80]. These signaling lipids illustrate how lipid metabolism can generate molecules that act as local or systemic signals.

### 5.4. Does Lipid Diversity Encode Cellular Information?

Taken together, these signaling systems raise an intriguing possibility: the large diversity of lipid species may allow membranes to encode regulatory information. Unlike nucleic acids, lipids do not form linear polymers that store information in a sequence. However, the combinatorial diversity of lipid species and their spatial organization within membranes may create a rich regulatory landscape that influences protein localization, enzymatic activity, and signaling network dynamics [64]. In this sense, lipid composition could function as a form of context-dependent molecular information that shapes cellular behavior. While this idea remains speculative, it highlights the possibility that lipid diversity may have functional significance beyond its contribution to membrane structure.

## 6. Lipid Remodeling and Dynamic Lipidomes

While the previous sections have focused on structural and signaling roles of lipids, another important feature of lipid biology is the dynamic nature of lipid metabolism. Cellular lipid composition is not static; instead, lipid molecules are continuously synthesized, modified, and degraded through interconnected metabolic pathways [18,37,81,82,83].

These metabolic processes generate a constantly changing lipid landscape within cellular membranes. Such dynamics suggest that lipid diversity may emerge not only from biosynthetic possibilities but also from the regulatory flexibility of lipid metabolic networks.

### 6.1. Acyl Chain Remodeling and the Lands Cycle

One of the major mechanisms contributing to lipid diversity is acyl chain remodeling, commonly referred to as the Lands cycle. In this process, phospholipids undergo cycles of deacylation and reacylation. Phospholipase enzymes remove a fatty acyl chain from a phospholipid, generating a lysophospholipid intermediate. This intermediate can then be reacylated by acyltransferases using a different fatty acid [17,18,23]. Through repeated cycles of remodeling, cells can modify the acyl chain composition of membrane lipids without altering their headgroup identity. This mechanism greatly expands the diversity of lipid species present in membranes. Importantly, remodeling processes are often selective with respect to fatty acid type. For example, certain phospholipids preferentially incorporate polyunsaturated fatty acids such as arachidonic acid or docosahexaenoic acid [23,37,72]. These fatty acids can later serve as precursors for lipid mediators, linking membrane composition to signaling pathways.

### 6.2. Lipid Turnover and Metabolic Flux

Another key feature of lipid metabolism is the relatively rapid turnover of many lipid species. Even structural phospholipids in cellular membranes can undergo continuous cycles of synthesis and degradation [6,11,23]. This turnover allows cells to adjust membrane composition in response to environmental conditions such as temperature, nutrient availability, or metabolic stress. For example, changes in fatty acid saturation levels can modulate membrane fluidity and maintain optimal membrane function under different physiological conditions [3,84]. Lipid metabolic flux therefore represents an important regulatory layer that controls the composition of the cellular lipidome.

### 6.3. Lipid Metabolism as a Regulatory Network

The complexity of lipid metabolism also suggests that lipid diversity may arise from the architecture of metabolic networks themselves. Lipid biosynthesis involves numerous enzymes that operate in interconnected pathways [85]. Changes in the activity of a single enzyme can influence the abundance of multiple lipid species simultaneously [3,18,82]. For example, alterations in fatty acid synthesis, desaturation, or elongation can affect the composition of many downstream lipid molecules. Similarly, phospholipid remodeling pathways can redistribute fatty acids among different lipid classes. This network structure implies that lipid diversity may reflect the system-level behavior of metabolic pathways rather than the specific function of individual lipid molecules.

### 6.4. Dynamic Lipidomes and Cellular Adaptation

Recent lipidomic studies have demonstrated that lipid composition can change substantially under different physiological conditions [86]. For example, lipidomes differ between cell types, developmental stages, and environmental conditions [6,12,87]. Cells also remodel their lipid composition in response to metabolic stress, nutrient availability, and signaling cues. These observations suggest that lipid diversity may enable cells to adapt membrane properties and signaling capacity to changing physiological demands. In this sense, the lipidome can be viewed as a dynamic molecular system that integrates metabolic activity with cellular regulation.

## 7. Evolutionary Perspectives on Lipid Diversity

Understanding why cells maintain thousands of lipid species may ultimately require an evolutionary perspective. Biological membranes are universal features of living cells, and the diversity of lipid molecules found in modern organisms likely reflects a long history of evolutionary adaptation [38,39,88].

Membranes must satisfy multiple functional requirements simultaneously: they must form stable permeability barriers, support membrane protein function, allow controlled transport of molecules, and remain adaptable to changing environmental conditions. These constraints may have shaped the evolution of membrane lipid composition across different domains of life [39,89,90].

### 7.1. The Archaeal–Bacterial Lipid Divide

One of the most striking examples of membrane lipid diversity arises from the fundamental differences between archaeal and bacterial membranes. Bacterial and eukaryotic membranes are primarily composed of glycerol-3-phosphate-based phospholipids with ester-linked fatty acids [91]. In contrast, archaeal membranes contain glycerol-1-phosphate backbones with ether-linked isoprenoid chains [38,39,92]. This structural distinction is so profound that it has been referred to as the “lipid divide” between Archaea and Bacteria [38,39]. Despite these chemical differences, both types of membranes perform similar biological functions. This observation suggests that multiple chemical solutions can satisfy the fundamental physical requirements of biological membranes.

### 7.2. Environmental Adaptation of Membrane Lipids

Another evolutionary driver of lipid diversity may be adaptation to environmental conditions. Membrane fluidity must be maintained within a relatively narrow range for proper cellular function. Organisms living in different environments often adjust their membrane lipid composition to maintain appropriate membrane properties. For example, changes in fatty acid saturation and chain length are commonly used to regulate membrane fluidity in response to temperature changes [26,93,94]. Extremophilic organisms provide striking examples of lipid adaptation [95]. Some archaea inhabiting high-temperature environments possess unique lipid structures that increase membrane stability under extreme conditions [38,96,97,98]. These observations suggest that lipid diversity may reflect evolutionary solutions to environmental challenges.

### 7.3. Organ-Specific Lipid Composition and Functional Specialization

In addition to global cellular constraints, lipid composition is also strongly shaped by organ- and tissue-specific functional requirements. Different organs exhibit characteristic lipid profiles that are highly conserved and tightly regulated, suggesting that lipid diversity is not merely stochastic but reflects specialized biological functions [3].

One of the most striking examples is the enrichment of highly polyunsaturated fatty acids, particularly docosahexaenoic acid (DHA), in the brain and retina. DHA-containing phospholipids contribute to membrane fluidity, curvature, and the function of membrane proteins involved in synaptic transmission and phototransduction. The selective accumulation of DHA in these tissues is not a passive consequence of lipid availability but is actively regulated through coordinated control of fatty acid uptake, transport, and remodeling pathways [99].

Another example is the accumulation of squalene in the skin, where it contributes to barrier function, protection against oxidative stress, and maintenance of membrane integrity. This tissue-specific enrichment reflects the expression of specialized lipid biosynthetic enzymes and regulatory pathways adapted to the physiological roles of the skin [100].

These observations suggest that organ-specific lipid composition arises from tightly regulated metabolic networks rather than random variation. Tissue-specific expression of lipid metabolic enzymes, selective substrate utilization, and controlled lipid transport all contribute to the establishment of distinct lipidomes across organs [25,74,85,86].

From this perspective, lipid diversity is not only a consequence of general cellular constraints but also a reflection of functional specialization at the tissue level. Organ-specific lipidomes therefore provide an important layer of biological regulation, linking lipid metabolism to physiological function.

### 7.4. Evolution of Lipid Metabolic Networks

The complexity of modern lipidomes may also arise from the evolution of lipid metabolic pathways. Lipid biosynthesis involves numerous enzymes that catalyze fatty acid synthesis, elongation, desaturation, and headgroup modification. Gene duplication and divergence of these enzymes over evolutionary time could have expanded the range of lipid molecules produced by cells [26,101]. In addition, metabolic network architecture may favor the generation of multiple lipid species. Enzymes involved in lipid metabolism often exhibit partial substrate specificity, allowing them to process a range of related lipid molecules. This biochemical flexibility can generate diverse lipidomes even from relatively small sets of enzymes [3]. From this perspective, lipid diversity may be an emergent property of evolving metabolic networks.

### 7.5. Evolutionary Advantages of Lipid Diversity

If lipid diversity is maintained by natural selection, it must confer some evolutionary advantage. One possibility is that lipid diversity provides robustness in membrane function. A diverse lipid composition may allow membranes to maintain appropriate physical properties across a range of environmental conditions. Another possibility is that lipid diversity supports the regulation of membrane proteins and signaling pathways. If different lipid species interact selectively with different proteins, a diverse lipid environment could enable more flexible regulation of cellular processes. Finally, lipid diversity may facilitate the formation of heterogeneous membrane domains that organize cellular processes spatially. While these hypotheses remain incompletely tested, they highlight the possibility that lipid diversity represents an evolutionary strategy for maintaining functional flexibility in cellular membranes.

## 8. Toward a Unifying Framework for Lipid Diversity

The preceding sections have outlined several conceptual frameworks that may explain why cells contain thousands of lipid species. These include the physical requirements of membrane organization, lipid–protein interactions, lipid-mediated signaling, metabolic network dynamics, and evolutionary adaptation. While each of these frameworks provides valuable insights, their relative explanatory power and limitations must be critically evaluated.

Among these models, the physical constraints of membrane organization are supported by extensive experimental and biophysical evidence. Numerous studies have demonstrated that lipid composition directly influences membrane properties such as fluidity, thickness, curvature, and phase behavior [29,31]. These properties, in turn, affect essential cellular processes including membrane trafficking, fusion, and domain formation. However, while this framework explains why lipid diversity may be necessary to tune membrane properties, it does not fully account for the specificity of individual lipid species observed in biological systems.

Lipid–protein interactions provide a second layer of explanation with strong structural and functional support. High-resolution studies have shown that many membrane proteins interact selectively with specific lipid molecules, including cholesterol, phosphoinositides, and cardiolipin [21]. These findings suggest that lipid diversity may be required to support the functional heterogeneity of membrane proteins. Nevertheless, this model is inherently protein-centric and does not fully explain why cells maintain a much larger diversity of lipid species than the number of protein-specific interactions would seemingly require.

In contrast, lipid signaling frameworks offer clear functional roles for specific lipid molecules, particularly in pathways involving phosphoinositides, sphingolipids, and lipid-derived second messengers [22]. These systems demonstrate that certain lipid species act as highly specific regulatory molecules. However, signaling lipids typically represent a relatively small fraction of the total lipidome and therefore cannot, on their own, explain the large-scale diversity observed in cellular membranes.

Metabolic network models provide a complementary perspective by emphasizing the role of biosynthetic pathways and lipid remodeling processes. The combinatorial nature of fatty acid synthesis, elongation, desaturation, and remodeling can generate a large number of lipid species as an emergent property of the system [74]. While this framework explains how lipid diversity can arise, it does not necessarily explain why such diversity is maintained under selective pressure.

Finally, evolutionary perspectives suggest that lipid diversity reflects long-term adaptation to environmental and functional constraints [95]. Comparative studies across organisms indicate that membrane lipid composition has been shaped by factors such as temperature, energy metabolism, and cellular architecture. However, evolutionary explanations are often difficult to test directly and may lack mechanistic specificity at the cellular level.

Taken together, these considerations indicate that no single framework is sufficient to explain lipid diversity. Instead, lipid diversity arises from the integration of multiple constraints operating at different biological scales. In this context, the physical constraints of membrane organization can be considered the primary driving force, as they impose fundamental requirements on membrane stability, fluidity, thickness, and curvature. Lipid–protein interactions provide a second layer of functional specificity, enabling selective regulation of membrane proteins. Lipid signaling systems add a regulatory layer, in which specific lipid species act as signaling mediators in spatially and temporally controlled processes. In parallel, lipid metabolic networks serve as generative systems that produce lipid diversity through combinatorial biosynthesis and remodeling. Finally, evolutionary processes act as a selection filter, shaping lipid composition over long timescales to optimize membrane function under environmental and physiological constraints.

## 9. Future Directions

Despite major advances in lipidomics and membrane biology, many fundamental questions about lipid diversity remain unresolved.

For example:

- Do individual lipid species have distinct functional roles, or are most lipid molecules functionally interchangeable?
- How do lipid metabolic networks generate and maintain characteristic lipid compositions in different organelles?
- To what extent does lipid composition regulate membrane protein activity and cellular signaling?
- How has lipid diversity evolved across different domains of life?

Addressing these questions will require integrating approaches from lipidomics, structural biology, membrane biophysics, and systems biology.

As new technologies continue to improve our ability to measure and manipulate lipid composition, future studies may reveal fundamental principles that explain why cells maintain such diverse lipid repertoires.

## Figures and Tables

**Table 1:** Hypotheses Proposed to Explain Lipid Diversity in Cells.

Conceptual Hypothesis	Key Concept	Biological Mechanism	Supporting Evidence	Key References
Membrane physical constraints	Lipid diversity tunes membrane physical properties	Lipid composition controls membrane fluidity, thickness, curvature, phase behavior	Membrane biophysics studies; phase separation; cholesterol effects	[2,29,30,31]
Lipid–protein interactions	Membrane proteins require specific lipid environments	Annular lipids, non-annular lipid binding, hydrophobic matching	Structural studies of membrane proteins; lipid binding pockets	[21,32,33]
Lipid signaling systems	Lipids function as signaling molecules	Phosphoinositides, DAG, sphingolipid signaling	PIP_2_ signaling; PKC activation; S1P signaling	[22,34,35,36]
Lipid metabolic network dynamics	Remodeling and metabolic flux generate diversity	Lands cycle; acyl chain remodeling; lipid turnover	Lipidomic studies showing dynamic lipidomes	[17,18,23,37]
Evolutionary adaptation	Membrane lipids evolved to meet environmental constraints	Archaeal–bacterial lipid divide; environmental adaptation	Comparative lipidomics and membrane evolution	[25,38,39]

**Table 2:** Structural Sources of Lipid Diversity.

Structural Variable	Example Lipid Classes	Functional Consequences	Key References
Headgroup composition	PC, PE, PS, PI	Surface charge, protein recruitment	[1,13]
Acyl chain length	C14–C24 fatty acids	Membrane thickness	[30,31]
Degree of unsaturation	Saturated vs. polyunsaturated lipids	Membrane fluidity, phase behavior	[3]
Lipid backbone	Glycerol vs. sphingoid base	Packing and membrane order	[1]
Lipid remodeling	Lands cycle modifications	Dynamic lipidome composition	[17,18]

**Table 3:** Examples of Lipids with Well-Defined Regulatory Functions.

Lipid	Major Cellular Role	Mechanism	Key References
PIP_2_	Membrane signaling	Recruitment of PH-domain proteins	[34,35]
DAG	Second messenger	Activation of PKC	[22]
Ceramide	Stress signaling	Regulation of apoptosis pathways	[22]
S1P	Extracellular signaling	GPCR activation	[36]
Cardiolipin	Mitochondrial membrane organization	Stabilization of respiratory chain complexes	[61]

## Data Availability

No new data were created or analyzed in this study. Data sharing is not applicable to this article.

## Abbreviations

DAG	Diacylglycerol
DHA	Docosahexaenoic acid
GPCR	G protein-coupled receptor
IP3	Inositol 1,4,5-trisphosphate
LPA	Lysophosphatidic acid
PC	Phosphatidylcholine
PE	Phosphatidylethanolamine
PH	Pleckstrin homology
PI	Phosphatidylinositol
PIP_2_	Phosphatidylinositol 4,5-bisphosphate
PKC	Protein kinase C
PLC	Phospholipase C
PS	Phosphatidylserine
PX	Phox homology
S1P	Sphingosine-1-phosphate
SMase	Sphingomyelinase
